# A Study of the Relationship Between Serum Albumin–Corrected Fructosamine and Type 2 Diabetic Retinopathy

**DOI:** 10.1155/jdr/9275699

**Published:** 2026-02-02

**Authors:** Zejiang Liu, Qiyun Long, Xuhui Song, Qin Guo, Tao Li, Huaguo Wang, Xing Qi, Sheng Lin

**Affiliations:** ^1^ Department of Laboratory Medicine, Ziyang Central Hospital, Ziyang, Sichuan Province, China; ^2^ Department of Ophthalmology, Ziyang Central Hospital, Ziyang, Sichuan Province, China

**Keywords:** albumin-corrected fructosamine, diabetic retinopathy, fructosamine, serum albumin, Type 2 diabetes

## Abstract

**Objective:**

To examine the association between albumin‐corrected fructosamine (AlbF) levels and the presence of diabetic retinopathy (DR) in adults with Type 2 diabetes mellitus (T2DM).

**Methods:**

This cross‐sectional study retrospectively analyzed 1263 inpatients with T2DM. After applying exclusion criteria, 415 patients were included and categorized into DR (*n* = 174) and non‐DR (*n* = 241) groups based on fundus examination. The association between the AlbF—analyzed both continuously (per 10 *μ*mol/g increment) and categorically (by tertiles)—and DR was assessed using multivariable logistic regression with progressive adjustment for sociodemographic, clinical, and laboratory confounders. Supplementary analyses, including receiver operating characteristic (ROC) curve assessment and interaction testing across predefined subgroups, evaluated the association′s robustness and consistency.

**Results:**

The prevalence of DR was 41.9%. Following full adjustment, each 10 *μ*mol/g increment in AlbF was associated with higher odds of having DR (adjusted OR = 1.88, 95% CI: 1.36–2.60; *p* < 0.001). A significant dose–response relationship was observed (*p* for trend < 0.001), with patients in the highest AlbF tertile exhibiting 7.20 times the odds of DR (95% CI: 2.82–18.40) compared to the lowest tertile. Furthermore, the association remained consistent across all predefined subgroups (*p* for interaction > 0.05 for all).

**Conclusions:**

Elevated AlbF was independently associated with the presence of DR in adults with T2DM, demonstrating a significant dose–response relationship. AlbF shows promise as a biomarker candidate for DR identification and stratification. Its potential clinical utility requires validation in larger prospective studies.

**Summary**

• **What Is Already Known?** Serum albumin–corrected fructosamine (AlbF) is a biomarker that reflects medium‐term glycemic control while correcting for variations in serum albumin. However, its association with diabetic retinopathy (DR) in patients with Type 2 diabetes has not been clearly established.

• **What This Study Has Found?** In this cross‐sectional study of 415 inpatients, we found that elevated AlbF levels were independently associated with higher odds of DR after comprehensive multivariable adjustment. Per 10 *μ*mol/g increase was associated with a 1.88‐fold higher odd of DR, and a significant dose–response relationship was observed. This association was robust across extensive sensitivity and subgroup analyses.

• **What Are the Implications of the Study?** AlbF may serve as a novel and promising biomarker for medium‐term glycemic monitoring. Our findings provide evidence linking AlbF to DR risk, generating the hypothesis that AlbF could potentially aid in risk stratification for DR. This warrants further investigation in prospective cohorts to validate its clinical utility.

## 1. Introduction

According to the International Diabetes Federation (IDF), 454 million people worldwide will have diabetes mellitus (DM) by 2030, and this number is projected to reach 700 million by 2045 [1]. In China, the prevalence of diabetes has grown rapidly, with authoritative estimates suggesting that the number of affected individuals reached approximately 140 million by 2022, posing a major public health challenge. Diabetic retinopathy (DR) is one of the most common microvascular complications of diabetes and remains a leading cause of preventable blindness among middle‐aged and older adults [2]. With increasing diabetes prevalence, the global burden of DR is also projected to rise substantially, highlighting the need for improved risk assessment strategies. Previous studies have reported that risk factors for DR can be broadly classified as controllable or uncontrollable, with glycemic control being a key modifiable factor [3]. Furthermore, glucose variability has been associated with retinopathy in patients with Type 2 diabetes [4]. Fructosamine (FMN), which reflects mean blood glucose levels over the preceding 2–3 weeks, provides an alternative to HbA1c for short‐term glycemic assessment. However, its accuracy is influenced by serum albumin concentrations [5]. To address this limitation, albumin‐corrected fructosamine (AlbF) has been proposed as a more robust measure than uncorrected FMN [6–10]. AlbF has been investigated in diverse contexts, including the diagnosis of diabetes, evaluation of glycemic control in patients with diabetes and those receiving peritoneal dialysis, and prediction of all‐cause and noncardiovascular mortality in non‐diabetic populations [11–13]. Despite these findings, the relationship between AlbF and DR in Type 2 diabetes remains poorly understood. Only limited studies have examined this association, and their results have been inconclusive. Therefore, this study was aimed at retrospectively investigating the association between AlbF and the presence of DR in patients with Type 2 diabetes, in order to provide preliminary evidence for its potential utility in clinical risk stratification.

## 2. Materials and Methods

### 2.1. Study Population and Design

**Figure 1:**
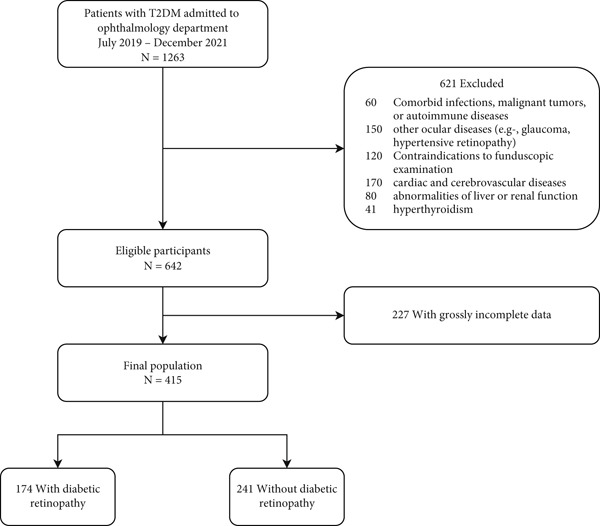
Participant flow in the AlbF‐DR risk assessment study.

This retrospective study initially screened a total of 1263 patients with Type 2 diabetes mellitus (T2DM) who were admitted to the Ophthalmology Department of Ziyang Central Hospital between January 2019 and December 2021. These patients were hospitalized for evaluation and management of various ophthalmologic conditions. After applying the exclusion criteria (detailed below), 415 patients remained eligible and were included in the final analysis (Figure 1). The process of participant screening, exclusion (with justifications), and eventual inclusion is summed up in this diagram that complies with Strengthening the Reporting of Observational Studies in Epidemiology (STROBE). We deliberately applied rigorous screening to minimize potential confounding by comorbidities and to ensure that the study population represented a clinically relevant cohort. Among the 415 patients, the presence or absence of DR was determined by retinal examination. To enhance diagnostic accuracy, all retinal assessments were conducted by two experienced ophthalmologists independently, and discrepancies were resolved by consensus. Patients were subsequently categorized into two groups: nondiabetic retinopathy (NDR; *n* = 241) and DR (*n* = 174). The NDR group included 89 males and 152 females, mean age 67.6 ± 9.0 years; the DR group included 55 males and 119 females, mean age 57.9 ± 9.8 years.

### 2.2. Inclusion and Exclusion Criteria

The inclusion criteria were as follows: (1) fulfilment of the clinical diagnostic criteria for T2DM [14] and (2) confirmation and staging of DR: dilated pupils and fundusography by a professional ophthalmologist. Subjects were considered to have DR if either eye showed the following lesions: microaneurysms, cotton wool spots, hemorrhages, intraretinal microvascular abnormalities, venous beading, hard exudates, or neovascularization [15].

Exclusion criteria were as follows: (1) combination of infections, malignant tumors, autoimmune diseases; (2) combination of ocular diseases such as glaucoma and hypertensive retinopathy; (3) combination of abnormalities of liver and renal function; (4) cardiac and cerebrovascular diseases; (5) contraindications to funduscopic examination; (6) hyperthyroidism; and (7) grossly incomplete data.

### 2.3. Data Collection

Clinical and laboratory data were retrieved from the hospital′s electronic medical records and Laboratory Information System (LIS). The collected data encompassed the following categories: demographic and anthropometric characteristics: sex, age, and body mass index (BMI); behavioral factors: smoking status (nonsmoker or current smoker) and alcohol consumption (nondrinker or current drinker); medical history: duration of diabetes; history of hypertension; medication use: detailed records of current glucose‐lowering and antihypertensive agents; laboratory parameters: complete blood count: white blood cell count, red blood cell count, hemoglobin concentration, and platelet count; comprehensive metabolic profile: total serum protein, serum albumin, FMN, glycated hemoglobin (HbA1c), blood creatinine, blood uric acid, lipid profile (total cholesterol, triglycerides, high‐density lipoprotein cholesterol (HDL‐C), and low‐density lipoprotein cholesterol (LDL‐C)).

The study was approved by the Ethics Committee of Ziyang Central Hospital, Sichuan Province (202083), and informed written consent was obtained from each patient prior to inclusion.

### 2.4. Laboratory Measurements

Blood samples were collected from each participant following a standard protocol. Two 2‐mL tubes of EDTA‐anticoagulated whole blood were drawn for complete blood count analysis and HbA1c detection. These were analyzed using a Sysmex XN‐1000 automated hematology analyzer and a Bio‐Rad glycated hemoglobin analyzer, respectively. Additionally, a 2‐mL blood sample was collected into a serum separation tube, allowed to clot, and centrifuged at 4000 rpm for 10 min to obtain serum. The serum was used to measure biochemical indicators, including liver and renal function markers, serum albumin, and FMN. These analyses were performed on a Siemens ADVIA 2400 automated clinical chemistry analyzer using reagents and calibrators from Sichuan Mack Co., Ltd. Serum albumin was measured by the bromocresol green method and FMN by the nitrotetrazolium blue method. The AlbF ratio was calculated using the following formula: AlbF (*μ*mol/g) = FMN (*μ*mol/L)/serum albumin (g/L).

## 3. Statistical Analysis

### 3.1. Handling of Missing Data

The prevalence of missing data for each variable is summarized in Table S1. As the proportion of missingness was below 20% for all covariates included in the models, missing data were addressed using multiple imputation by chained equations (MICE) to generate five imputed datasets. Analysis results from these datasets were pooled according to Rubin′s rules. A complete‐case analysis was performed as a sensitivity analysis to verify the robustness of the primary findings. The final analytic sample size of 415 refers to the imputed pooled dataset generated through multiple imputation by MICE.

### 3.2. Primary Analysis

Multivariable logistic regression was employed to quantify the association between AlbF and DR, expressed as odds ratios (ORs) with 95% confidence intervals (CIs). AlbF was analyzed both as a continuous variable (per 10 *μ*mol/g increment) and as a categorical variable (tertiles: T1, T2, and T3). A trend test was performed across AlbF tertiles to evaluate dose–response relationships. Multivariable regression models employed sequential adjustment:

• Model 1: crude (unadjusted association)
• Model 2: demographic (adjusted for age and sex)
• Model 3 (core clinical): Model 2 plus diabetes duration, use of glucose‐lowering medication, hypertension, and BMI—variables reflecting disease severity and established DR risk factors
• Model 4: full model (Model 3 + HbA1c [glycemic control], creatinine [renal function], total cholesterol, triglycerides [lipid metabolism], lymphocyte count [inflammation/immune status], and hemoglobin [oxygen transport])

### 3.3. Evaluation of the Albumin Correction and Model Diagnostics

To validate the use of the AlbF ratio, we compared its discriminative performance for DR against its individual components (FMN alone, albumin alone, and both included simultaneously) by calculating and comparing the area under the receiver operating characteristic curve (AUC) (Table S2 and Figure S1).

Multicollinearity among all covariates in the fully adjusted model was assessed using variance inflation factors (VIFs) and visualized through a correlation matrix diagram; all VIF values were below 4, indicating no substantial collinearity (Table S3; correlation matrix in Figure S2). The linearity of the association between continuous AlbF and the log‐odds of DR was examined using restricted cubic splines with four knots (Figure S3).

### 3.4. Subgroup Analyses and Effect Modification

**Figure 2:**
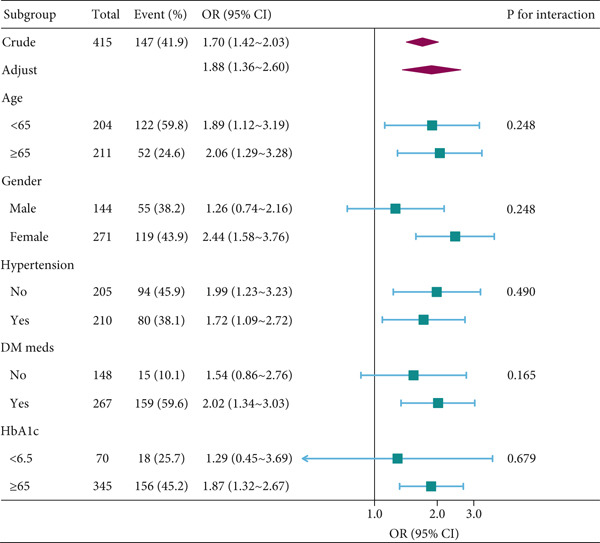
Association between AlbF(per 10 *μ*mol/g) and diabetic retinopathy according to the general characteristics. Except for the stratification factor itself, the stratifications were adjusted for the full model (age, gender, DM duration, DM meds, hypertension, BMI, glycated hemoglobin, creatinine, total cholesterol, triglycerides, lymphocyte, and hemoglobin). Abbreviations: AlbF, fructosamine/albumin; DR, diabetic retinopathy; DM, diabetes mellitus; DM duration, years since diagnosis; DM meds, use of glucose‐lowering or antihypertensive medication; BMI, body mass index.

Stratified analyses were performed to assess whether the association between AlbF (per 10 *μ*mol/g) and DR was consistent across prespecified subgroups: age (< 65 vs. ≥ 65 years), sex, hypertension status, use of glucose‐lowering medication, and HbA1c level (< 6.5% [48 mmol/mol] vs. ≥ 6.5% [48 mmol/mol]). The significance of effect modification (interaction) for each subgroup was tested by introducing a multiplicative interaction term into the fully adjusted model (Model 4) and comparing models using likelihood ratio tests (Figure 2).

**Figure 3:**
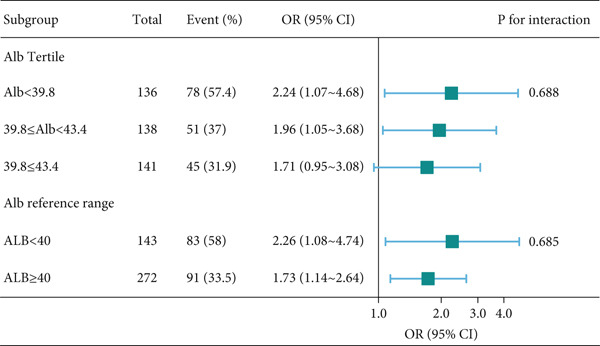
Forest plot of the association between AlbF (per 10 *μ*mol/g) and diabetic retinopathy, stratified by serum albumin levels. Tertile splits: ALB < 39.8 g/L, 39.8 g/L < ALB < 43.4 g/L, ALB ≥ 43.4 g/L. Clinical cut‐off: ALB < 40 g/L vs. ALB ≥ 40 g/L (WS/T 404.2‐2012, Chinese industry standard). Except for the stratification factor itself, the stratifications were adjusted for the full model (age, gender, DM duration, DM meds, hypertension, BMI, glycated hemoglobin, creatinine, total cholesterol, triglycerides, lymphocyte, and hemoglobin). Abbreviations: AlbF, fructosamine/albumin; DR, diabetic retinopathy; DM, diabetes mellitus; DM duration, years since diagnosis; DM meds, use of glucose‐lowering medication; BMI, body mass index.

Additionally, to evaluate the fundamental rationale for albumin correction, we performed a stratified analysis by serum albumin levels (using both tertiles and a clinical cut‐off of 40 g/L) to test the consistency of the AlbF‐DR association (Figure 3).

Statistical analyses were performed using R software Version 4.2.1 (R Foundation for Statistical Computing, Vienna, Austria), the R survey package Version 4.1‐1, and Free Statistics software Version 1.7.1 (Beijing FreeClinical Medical Technology Co., Ltd.). *p* < 0.05 (two‐tailed) [16] was declared significant.

## 4. Results

### 4.1. Baseline Characteristics of the Study Population

After applying the exclusion criteria and multiple imputation for missing data, a total of 415 patients were included in the final analysis. The baseline characteristics of the patients, stratified by the presence of DR, are presented in Table 1. The overall prevalence of DR was 41.9% (174/415). Compared to patients without DR (NDR group), those with DR were significantly younger (57.9 ± 9.8 vs. 67.6 ± 9.0 years, p<0.001), had a longer median duration of diabetes (10.0 (IQR 7.0–12.0) vs. 4.0 (IQR 1.0–10.0) years, *p* < 0.001), and had a higher prevalence of glucose‐lowering medication use (91.4% vs. 44.8%, *p* < 0.001). Additionally, the DR group exhibited significantly higher levels of HbA1c, FMN, and AlbF, but lower levels of serum albumin, red blood cell count, and hemoglobin (all *p* < 0.05).

**Table 1:** Baseline characteristics of the study population (after multiple imputation).

**Variables**	**Total (** **n** = 415**)**	**NDR (** **n** = 241**)**	**DR (** **n** = 174**)**	**p** **value**
Demographics				
Age (year)	63.5 ± 10.5	67.6 ± 9.0	57.9 ± 9.8	< 0.001
Gender, *n* (%)				0.261
Male	144 (34.7)	89 (36.9)	55 (31.6)	
Female	271 (65.3)	152 (63.1)	119 (68.4)	
BMI (kg/m^2^)	24.8 ± 3.6	25.0 ± 3.8	24.4 ± 3.3	0.122
Weight (kg)	60.0 ± 10.4	60.1 ± 10.9	59.9 ± 9.8	0.874
Height (cm)	155.5 ± 8.3	154.9 ± 8.6	156.4 ± 7.8	0.064
SBP (mmHg)	140.0 ± 21.4	140.8 ± 19.5	138.9 ± 23.8	0.357
DBP (mmHg)	80.1 ± 11.4	79.9 ± 10.2	80.4 ± 12.8	0.619
Smoking, *n* (%)				0.775
Never	395 (95.2)	230 (95.4)	165 (94.8)	
Ever	20 (4.8)	11 (4.6)	9 (5.2)	
Drinking, *n* (%)				0.026
Never	397 (95.7)	226 (93.8)	171 (98.3)	
Ever	18 (4.3)	15 (6.2)	3 (1.7)	
Laboratory measurements				
AlbF, per 10 *μ*mol/g ^∗^	4.9 ± 1.2	4.6 ± 1.2	5.3 ± 1.1	<0.001
Glucose (mmol/L)	8.1 ± 3.2	8.1 ± 3.1	8.0 ± 3.3	0.739
Glycated hemoglobin (%)	8.5 ± 2.1	8.2 ± 2.1	8.8 ± 2.0	0.004
Glycated hemoglobin (mmol/mol)	69.0 ± 22.4	66.3 ± 22.6	72.7 ± 21.7	0.004
Fructosamine (mmol/L)	2.0 ± 0.5	1.9 ± 0.5	2.1 ± 0.4	< 0.001
Total cholesterol (mmol/L)	5.2 ± 1.1	5.1 ± 1.0	5.3 ± 1.3	0.077
Triglycerides (mmol/L)	1.5 (1.0, 2.3)	1.5 (1.1, 2.2)	1.4 (1.0, 2.5)	0.958
HDL‐C (mmol/L)	1.5 ± 0.4	1.5 ± 0.4	1.6 ± 0.5	0.226
LDL‐C (mmol/L)	2.9 ± 0.9	2.8 ± 0.8	2.9 ± 0.9	0.606
Creatinine (*μ*mol/L)	61.4 ± 19.7	59.3 ± 18.0	64.4 ± 21.5	0.009
Urea (mmol/L)	6.0 ± 1.8	5.9 ± 1.7	6.1 ± 2.0	0.333
Uric acid (*μ*mol/L)	325.1 ± 89.2	319.0 ± 94.2	333.5 ± 81.3	0.101
Total protein (g/L)	67.1 ± 7.0	67.7 ± 7.3	66.2 ± 6.6	0.037
Albumin (g/L)	41.3 ± 4.3	42.3 ± 3.5	40.0 ± 4.8	<0.001
Prealbumin (g/L)	238.5 ± 44.0	240.2 ± 44.0	236.0 ± 44.2	0.343
White blood cell (10^9^/L)	6.0 ± 1.4	6.0 ± 1.3	6.1 ± 1.6	0.283
Neutrophil (10^9^/L)	3.6 ± 1.1	3.6 ± 1.1	3.6 ± 1.2	0.82
Lymphocyte (10^9^/L)	1.8 ± 0.6	1.8 ± 0.5	1.9 ± 0.6	0.041
Monocyte (10^9^/L)	0.4 ± 0.1	0.4 ± 0.2	0.4 ± 0.1	0.888
Red blood cell (10^12^/L)	4.3 ± 0.5	4.4 ± 0.5	4.2 ± 0.6	< 0.001
Hemoglobin (g/L)	129.5 ± 17.5	133.2 ± 16.3	124.5 ± 17.8	< 0.001
RDW‐SD (fL)	43.6 ± 3.7	44.1 ± 3.9	43.0 ± 3.3	0.002
RDW‐CV (%)	13.3 ± 1.2	13.3 ± 1.2	13.3 ± 1.2	0.609
Platelet (10^9^/L)	177.9 ± 58.3	174.9 ± 55.3	182.1 ± 62.1	0.21
Comorbidities and medications				
DM duration (years)	8.0 (2.0, 10.0)	4.0 (1.0, 10.0)	10.0 (7.0, 12.0)	< 0.001
DM meds, *n* (%)				< 0.001
No	148 (35.7)	133 (55.2)	15 (8.6)	
Yes	267 (64.3)	108 (44.8)	159 (91.4)	
Hypertension, *n* (%)				0.109
No	205 (49.4)	111 (46.1)	94 (54.0)	
Yes	210 (50.6)	130 (53.9)	80 (46.0)	
HTN duration (years)	4.0 (2.0, 10.0)	5.0 (2.0, 10.0)	3.0 (1.0, 10.0)	0.117
HTN meds, *n* (%)				0.294
No	61 (29.2)	34 (26.6)	27 (33.3)	
Yes	148 (70.8)	94 (73.4)	54 (66.7)	

*Note:* The analytic sample size of *n* = 415 refers to the imputed pooled dataset generated by multiple imputation by chained equations, not the raw complete‐case sample.

Abbreviations: AlbF, fructosamine/albumin; BMI, body mass index; DBP, diastolic blood pressure; DM, diabetes mellitus; DM duration/HTN duration, years since diagnosis; DM meds/HTN meds, use of glucose‐lowering or antihypertensive medication; DR, diabetic retinopathy; HDL‐C, high‐density lipoprotein cholesterol; LDL‐C, low‐density lipoprotein cholesterol; NDR, nondiabetic retinopathy; RDW‐CV, coefficient of variation of red blood cell distribution width; RDW‐SD, standard deviation of red blood cell distribution width; SBP, systolic pressure.

^∗^AlbF was entered as a continuous variable scaled per 10 *μ*mol/g to facilitate interpretation.

### 4.2. Univariable Logistic Regression Analysis

Table 2 presents the univariable logistic regression results for factors associated with DR. Mirroring the baseline comparisons, older age and higher hemoglobin levels were protective factors, whereas longer diabetes duration, use of glucose‐lowering medication, and elevated levels of HbA1c, creatinine, and AlbF were significantly associated with increased DR odds. Specifically, each 10 *μ*mol/g increment in AlbF demonstrated a strong association with DR (unadjusted OR = 1.70; 95% CI: 1.42–2.03; *p* < 0.001).

**Table 2:** Univariable logistic regression for diabetic retinopathy.

**Variable**	**OR (95% CI)**	**p** **value**
Demographics		
Age, year	0.90 (0.88~0.92)	< 0.001
Gender, *n* (%)	1.27 (0.84~1.91)	0.262
BMI (kg/m^2^)	0.96 (0.91~1.01)	0.123
Weight (kg)	1.00 (0.98~1.02)	0.874
Height (cm)	1.02 (1.00~1.05)	0.065
SBP (mmHg)	1.00 (0.99~1.00)	0.356
DBP (mmHg)	1.00 (0.99~1.02)	0.618
Smoking, *n* (%)	1.14 (0.46~2.81)	0.775
Drinking, *n* (%)	0.26 (0.08~0.93)	0.038
Laboratory measurements		
AlbF, per 10 *μ*mol/g ^∗^	1.70 (1.42~2.03)	< 0.001
Glucose (mmol/L)	0.99 (0.93~1.05)	0.739
Glycated hemoglobin (%)	1.15 (1.04~1.27)	0.005
Glycated hemoglobin (mmol/mol)	1.01 (1.00~1.02)	0.005
Fructosamine (mmol/L)	1.00 (1.00~1.00)	< 0.001
Total cholesterol (mmol/L)	1.17 (0.98~1.39)	0.078
Triglycerides (mmol/L)	1.06 (0.94~1.19)	0.357
HDL‐C (mmol/L)	1.32 (0.84~2.06)	0.226
LDL‐C (mmol/L)	1.06 (0.85~1.32)	0.605
Apolipoprotein A (g/L)	0.75 (0.35~1.63)	0.468
Apolipoprotein B (g/L)	1.92 (0.96~3.82)	0.064
Creatinine (*μ*mol/L)	1.01 (1.00~1.02)	0.01
Urea (mmol/L)	1.05 (0.95~1.17)	0.333
Uric acid (*μ*mol/L)	1.00 (1.00~1.00)	0.102
Total protein (g/L)	0.97 (0.94~1.00)	0.043
Albumin (g/L)	0.87 (0.83~0.92)	< 0.001
Prealbumin (g/L)	1.00 (0.99~1.00)	0.342
White blood cell (10^9^/L)	1.08 (0.94~1.24)	0.283
Neutrophil (10^9^/L)	1.02 (0.86~1.21)	0.819
Lymphocyte (10^9^/L)	1.45 (1.01~2.07)	0.042
Monocyte (10^9^/L)	0.91 (0.24~3.46)	0.888
Red blood cell (10^12^/L)	0.51 (0.35~0.75)	0.001
Hemoglobin (g/L)	0.97 (0.96~0.98)	< 0.001
RDW‐SD (fL)	0.92 (0.87~0.97)	0.003
RDW‐CV (%)	0.96 (0.82~1.13)	0.609
Platelet (10^9^/L)	1.00 (1.00~1.01)	0.21
Comorbidities and medications		
DM duration (years)	1.10 (1.07~1.14)	< 0.001
DM meds, *n* (%)	13.05 (7.26~23.48)	< 0.001
Hypertension, *n* (%)	0.73 (0.49~1.07)	0.11
HTN duration (years)	0.98 (0.93~1.04)	0.517
HTN meds, *n* (%)	0.72 (0.39~1.33)	0.295

Abbreviations: AlbF, fructosamine/albumin; BMI, body mass index; CI, confidence interval; DBP, diastolic blood pressure; DM, diabetes mellitus; DM duration/HTN duration, years since diagnosis; DM meds/HTN meds, use of glucose‐lowering or antihypertensive medication; HDL‐C, high‐density lipoprotein cholesterol; LDL‐C, low‐density lipoprotein cholesterol; OR, odds ratio; RDW‐CV, coefficient of variation of red blood cell distribution width; RDW‐SD, standard deviation of red blood cell distribution width; SBP, systolic pressure.

^∗^AlbF was entered as a continuous variable scaled per 10 *μ*mol/g to facilitate interpretation.

### 4.3. Multivariable Analysis of the Association Between AlbF and DR

The association between the AlbF and DR was further examined using multivariable logistic regression models with progressive adjustment for potential confounders (Table 3). When analyzed as a continuous variable (per 10 *μ*mol/g increment), the positive association between AlbF and DR remained persistently significant across all models. In the fully adjusted model (Model 4—which included age, sex, diabetes duration, use of glucose‐lowering or antihypertensive medication, hypertension, BMI, and key hematological and metabolic parameters HbA1c, creatinine, total cholesterol, triglycerides, lymphocyte count, and hemoglobin)—each 10 *μ*mol/g increase in AlbF was associated with an adjusted OR for DR of 1.88 (95% CI: 1.36–2.60; *p* < 0.001).

**Table 3:** Multivariable logistic regression results for serum AlbF and diabetic retinopathy.

**Variable**	**n.total**	**n.event %**	**Model 1**		**Model 2**		**Model 3**		**Model 4**	
			**Crude.OR (95% CI)**	**Crude** **p**	**Adj.OR (95% CI)**	**Adj** **p**	**Adj.OR (95% CI)**	**Adj** **p**	**Adj.OR (95% CI)**	**Adj** **p**
Continuous, per 10 *μ*mol/g∗	415	174 (41.9)	1.7 (1.42~2.03)	< 0.001	1.62 (1.34~1.97)	< 0.001	1.73 (1.37~2.18)	< 0.001	1.88 (1.36~2.60)	< 0.001
AlbF category (per 10 *μ*mol/g)										
T1 (low)	138	27 (19.6)	1 (Ref)		1 (Ref)		1 (Ref)		1 (Ref)	
T2	138	68 (49.3)	3.99 (2.33~6.83)	< 0.001	4.21 (2.31~7.68)	< 0.001	4.38 (2.20~8.73)	< 0.001	5.50 (2.53~11.96)	< 0.001
T3 (high)	139	79 (56.8)	5.41 (3.16~9.27)	< 0.001	5.24 (2.87~9.57)	< 0.001	5.45 (2.72~10.92)	< 0.001	7.20 (2.82~18.40)	< 0.001
*p* for trend				< 0.001		< 0.001		< 0.001		< 0.001

*Note:* Model1: unadjusted. Model2: adjusted for age and gender. Model3: Model 2 + DM duration, DM meds, hypertension, and BMI. Model4: Model 3 + glycated hemoglobin, creatinine, total cholesterol, triglycerides, lymphocyte, and hemoglobin.

Abbreviations: AlbF, fructosamine/albumin; BMI, body mass index; CI, confidence interval; DM, diabetes mellitus; DM duration, years since diagnosis; DM meds, use of glucose‐lowering or antihypertensive medication; HDL‐C, high‐density lipoprotein cholesterol; LDL‐C, low‐density lipoprotein cholesterol; OR, odds ratio.

^∗^AlbF was entered as a continuous variable scaled per 10 *μ*mol/g to facilitate interpretation. The analytic sample size of *n* = 415 refers to the imputed pooled dataset generated by multiple imputation by chained equations, not the raw complete‐case sample.

When patients were categorized into AlbF tertiles, a clear dose–response relationship was observed (*p* for trend < 0.001). Compared with those in the lowest tertile (T1), patients in the middle (T2) and highest (T3) tertiles exhibited significantly higher odds of DR in the fully adjusted model (adjusted OR = 5.50, 95% CI: 2.53–11.96; and adjusted OR = 7.20, 95% CI: 2.82–18.40, respectively).These findings highlight that AlbF demonstrates a continuous risk gradient, and exploratory analyses suggested possible threshold effects; however, these require confirmation in larger, prospective studies before any clinical application. Comprehensive diagnostics confirmed the robustness of the final logistic model, including a binned residual plot evaluating goodness‐of‐fit (Figure S4), Influence diagnostics identifying potential outliers (Figure S5), and multicollinearity assessment verifying variable independence (Figure S6).Taken together, these results support the robustness of the observed AlbF–DR association and suggest it is unlikely to be fully explained by model misspecification or statistical artefacts.

### 4.4. Sensitivity Analyses

To assess the robustness of the AlbF‐DR association, we conducted several sensitivity analyses. First, subgroup analyses were performed based on predefined characteristics: age (< 65 vs. ≥ 65 years), sex, hypertension status, glucose‐lowering medication use, and HbA1c level. The association remained consistently significant across all subgroups without evidence of interaction (all *p* interaction > 0.05; Figure 2).

Second, to evaluate potential confounding by baseline albumin levels, analyses were stratified by serum albumin concentration (using tertiles and a clinical cut‐off of 40 g/L). The association persisted significantly within all strata, with no observed interaction (*p* interaction > 0.05; Figure 3).

Additionally, we performed a comprehensive sensitivity analysis by incorporating a broader set of covariates into our fully adjusted model; the results of this analysis are presented in Table S4.

Finally, a complete‐case analysis was conducted excluding patients with missing data (*n* = 348). This analysis yielded results consistent in direction with the primary multiple‐imputation analysis; however, effect estimates were marginally attenuated and narrowly missed conventional statistical significance in the fully adjusted model (adjusted OR = 1.63 per 10 *μ*mol/g AlbF; 95% CI: 0.98–2.69; *p* = 0.059) (Table 4).

**Table 4:** Sensitivity analysis (remove all missing covariates).

**Variable**	**n.total**	**n.event_%**	**Model1**		**Model2**		**Model3**		**Model4**	
			**Crude.OR (95% CI)**	**Crude** **p**	**Adj.OR (95% CI)**	**Adj** **p**	**Adj.OR (95% CI)**	**Adj** **p**	**Adj.OR (95% CI)**	**Adj** **p**
AlbF, per 10 *μ*mol/g ^∗^	348	122 (35.1)	1.45 (1.20~1.76)	< 0.001	1.43 (1.17~1.76)	0.001	1.84 (1.22~2.78)	0.004	1.63 (0.98~2.70)	0.059
AlbF category (per 10 *μ*mol/g)										
T1 (low)	126	23 (18.3)	1 (ref)		1 (ref)		1 (ref)		1 (ref)	
T2	116	51 (44)	3.51 (1.96~6.29)	< 0.001	3.46 (1.85~6.47)	< 0.001	3.61 (1.76~7.40)	< 0.001	4.64 (2.07~10.40)	< 0.001
T3 (high)	106	48 (45.3)	3.71 (2.05~6.70)	< 0.001	3.62 (1.91~6.87)	< 0.001	3.62 (1.75~7.48)	0.001	5.08 (1.93~13.38)	0.001
*p* for trend				< 0.001		< 0.001		0.001		0.001

*Note:* Model1: unadjusted. Model2: adjusted for age and gender. Model3: Model 2 + DM duration, DM meds, hypertension, and BMI. Model4: Model 3 + glycated hemoglobin, creatinine, total cholesterol, triglycerides, lymphocyte, and hemoglobin.

Abbreviations: AlbF, fructosamine/albumin; BMI, body mass index; CI, confidence interval; DM, diabetes mellitus; DM duration, years since diagnosis; DM meds, use of glucose‐lowering or antihypertensive medication; HDL‐C, high‐density lipoprotein cholesterol; LDL‐C, low‐density lipoprotein cholesterol; OR, odds ratio.

^∗^AlbF was entered as a continuous variable scaled per 10 *μ*mol/g to facilitate interpretation.

## 5. Discussion

In this study, elevated AlbF levels were significantly associated with the presence of DR in patients with T2DM (*p* < 0.001). This association remained robust after comprehensive adjustment for a wide range of demographic, clinical, and laboratory confounders. Furthermore, subgroup analyses stratified by age, sex, hypertension status, glucose‐lowering medication use, and HbA1c level showed no significant interactions (all *p* for interaction > 0.05), indicating the stability of the AlbF–DR association across these characteristics. The robustness of AlbF across multiple subgroups suggests that its utility is not restricted to specific patient subsets, but may have broader applicability in routine diabetes care. The consistency of our findings after multiple adjustments and subgroup analyses suggests that AlbF may be a promising biomarker candidate for identifying the presence of DR in patients with T2DM. However, whether AlbF‐guided management strategies improve DR outcomes cannot be determined from this cross‐sectional study, and prospective interventional research is needed.

Furthermore, the FMN assay technology has been continuously improved and refined since its inception, taking into account economy, speed and simplicity, as well as increased robustness and reliability of results [17–19]. FMN is a simple, rapid and economical assay. Despite the advantages of blood glucose control, it is one of the most important controllable risk factors for DR. Short‐term fluctuations in blood glucose have been linked to the presence and severity of DR in patients with Type 2 diabetes [3]. FMN refers to glycated serum proteins, predominantly glycated albumin, and reflects average glycaemia over the preceding 2–3 weeks. FMN may be used as an adjunctive marker of short‐term glycemic control when HbA1c or short‐term glucose measures are unreliable [17, 20].FMN is less affected by anemia, hemoglobin variants, or pregnancy, and correlates well with HbA1c in people with diabetes. For monitoring short‐term blood glucose, there are still shortcomings, and serum albumin levels affect FMN results [21]. Although our study excluded patients with severe hepatic or renal dysfunction, serum albumin levels can still vary substantially among individuals with Type 2 diabetes due to age, nutritional status, or comorbid conditions. Even within this relatively stable population, uncorrected FMN may be biased by such variation. AlbF, by normalizing FMN to albumin concentration, mitigates this influence and thus provides a more consistent and physiologically meaningful index of short‐term glycemic exposure. Previous studies have reported reduced serum albumin levels in patients with T2DM [22], which is more pronounced in those with DR [23], a finding consistent with our results. These findings support the rationale for considering AlbF as a potential alternative biomarker candidate in T2DM and DR patients, since uncorrected FMN results are affected by serum albumin and may not accurately reflect short‐term glycemic status. Poor glycaemic control is a recognized risk factor for DR. [24] Randomized trials (e.g., UKPDS) have demonstrated that intensive glycemic control can reduce the incidence of microvascular complications [25]. Taken together, these findings suggest that AlbF retains the advantages of FMN as a short‐term glycemic indicator, while potentially reducing bias related to albumin variability. This may provide complementary information to HbA1c, although its incremental clinical utility requires confirmation in future studies. The findings from this study suggest that AlbF holds promise as a complementary glycemic marker, particularly in clinical scenarios where HbA1c is known to be unreliable, such as in patients with anemia, hemoglobin variants, or altered red blood cell turnover [26–28]. In these situations, AlbF could provide a valuable short‐term index of glycemic control. While this study was not primarily designed to establish definitive clinical cut‐offs, our exploratory analysis identified a potential AlbF threshold of 38.0 *μ*mol/g, which corresponded with a sensitivity of 74% and specificity of 60%, yielding a Youden’′s index of 1.33, indicating moderate discriminatory performance (Figure S7). This observation warrants further investigation in prospectively designed cohorts to define and validate optimal diagnostic thresholds. From a practical standpoint, the derivation of AlbF from established and widely available assays for FMN and serum albumin enhances its translational potential. As these components are routinely measured in clinical biochemistry panels, AlbF can often be reported without additional costs, potentially offering a cost‐efficient alternative to more specialized assays. Future studies should focus on validating these thresholds in diverse populations and conducting formal cost‐effectiveness analyses to firmly establish the role of AlbF in routine diabetes management.

In the fully adjusted model, each 10 *μ*mol/g increase in AlbF corresponded to an adjusted OR of 1.88 (95% CI: 1.36–2.60) for DR. Restricted cubic spline analyses indicated a statistically significant departure from linearity (*p* for nonlinearity = 0.004), suggesting that the association may be nonlinear at higher AlbF levels, potentially indicative of a threshold effect. However, given the limited sample size at extreme AlbF values, the evidence for a specific threshold point remains inconclusive. Consequently, while this non‐linearity warrants further investigation, the linear model was retained as the primary analytical approach for parsimony and clinical interpretability.

The possible mechanisms underlying the observed AlbF–DR association may involve several pathways. First, persistent hyperglycemia has been linked to an increased risk of diabetic complications and may aggravate existing conditions through nonenzymatic glycation of plasma proteins [29], including albumin (which constitutes ~80% of FMN). Glycated albumin can stimulate cellular expression of cytokines such as TNF‐*α*, IL‐1*β*, and IL‐6 [30], which have been implicated in the pathophysiology of DR [31, 32]; second, advanced glycation end products (AGEs) contribute to diabetic vascular injury [33] and are thought to play a role in DR pathogenesis [34]. Collectively, these mechanisms provide a biological rationale for AlbF as more than a statistical correlate, but as a plausible marker reflecting both glycemic exposure and inflammatory activity. This dual role—capturing glycemic burden while simultaneously linking to inflammatory pathways—suggests that AlbF may have potential relevance for risk stratification in DR, though this requires further validation. AlbF may therefore have potential applications in short‐term glycemic assessment in T2DM and could be further explored for clinical relevance. However, whether AlbF‐guided management strategies improve DR outcomes cannot be determined from this cross‐sectional study, and prospective interventional research is needed. In patients with conditions that affect HbA1c accuracy (e.g., anemia or hemoglobin variants), FMN remains a feasible short‐term glycemic marker; nevertheless, it is influenced by serum protein concentrations, and AlbF helps mitigate this bias. This aligns with our statistical findings, which remained robust even after comprehensive adjustment for multiple covariates. Moreover, the persistence of the AlbF–DR association after adjusting for traditional risk factors such as BMI and hypertension suggests a potential additive role. The persistent positive association in HbA1c‐controlled patients aligns with the hypothesis that AlbF captures short‐term glycemic variability that escapes HbA1c′s 3‐month averaging window. Specifically, HbA1c has limited sensitivity to acute glucose excursions [35, 36], whereas FMN—with its shorter 2–3 week reflection window—may better capture postprandial hyperglycemia and short‐term glycemic variability [21]. These transient fluctuations can independently activate oxidative stress and inflammatory pathways implicated in retinal microvascular damage [37, 38]. Taken together, this pattern suggests that AlbF may help identify residual DR risk among apparently well‐controlled patients, potentially serving as an early indicator of subclinical injury or as a guide for refining glycemic management in those with optimal HbA1c. AlbF might complement existing risk models, but this possibility remains to be verified in future research. The AlbF–DR association persisted after adjustment for a wide range of clinical covariates, including BMI and antihypertensive medication use, supporting the robustness of our findings and the potential of AlbF as a biomarker candidate.

### 5.1. Limitations

This study has several limitations. First, its retrospective, cross‐sectional design precludes causal inference and cannot establish temporality; prospective studies are required. Second, some variables (e.g., diabetes duration and medication use) were self‐reported and may be subject to recall bias. Third, the generalizability of our findings may be limited by the single‐center, hospital‐based design. The relatively high prevalence of DR in our cohort suggests that our sample may overrepresent patients with more advanced disease, potentially limiting the direct extrapolation of our risk estimates to broader community‐based or primary care populations. Hospital‐based samples are frequently characterized by higher disease burden and longer diabetes duration compared with community cohorts [39–41], which may partially explain the relatively high prevalence of DR observed in our study. Thus, extrapolation to primary care or newly diagnosed populations should be cautious. Fourth, because of high missingness (≥ 50%) for variables such as type/duration of antihypertensive therapy, we adjusted only for hypertension diagnosis, which may incompletely capture treatment intensity. Finally, we did not stratify DR into nonproliferative versus proliferative subtypes. Future research with larger, multicenter prospective cohorts is needed to validate AlbF as a biomarker, to examine potential thresholds, and to explore whether AlbF‐guided management strategies could improve outcomes. In particular, prospective longitudinal studies should assess whether baseline AlbF predicts incident DR or the progression from nonproliferative to proliferative stages. Moreover, evaluating whether dynamic changes in AlbF provide incremental predictive value beyond HbA1c would be an important next step. Finally, testing AlbF within multivariable risk models and assessing its cost‐effectiveness in community‐based, multicenter populations will be essential for clinical translation.

In summary, AlbF levels were significantly higher in patients with T2DM and DR, and higher AlbF levels were consistently associated with greater odds of having DR after extensive covariate adjustment and subgroup analyses. These findings support AlbF as a potential biomarker candidate for DR identification, although its clinical utility for risk stratification or guiding interventions requires validation in larger, prospective, multicenter studies.

## Ethics Statement

Ethics approval was obtained from the Ziyang Central Hospital Ethics Review Committee(202083).

## Disclosure

All authors had access to the data. All authors read and approved the final manuscript.

## Conflicts of Interest

The authors declare no conflicts of interest.

## Author Contributions

Zejiang Liu: study design, data collection, and manuscript writing. Qiyun Long: analyzed and interpreted the data and results and drafted the manuscript. Xuhui Song, Qin Guo, Xing Qi, and Huaguo Wang: revised the manuscript. Tao Li: data collection and definitive diagnosis of diabetic retinopathy. Sheng Lin: proposed the concept and design of the study and revised the manuscript for critical intellectual content.

## Funding

This work was supported by the Science and Technology Innovation Base (Platform) Project of Ziyang, No. Zykjjsc20‐cxpt‐2020‐02 and Establishment of lncRNA spectrum and risk prediction model related to heart failure in hemodialysis participants in Sichuan–Chongqing region based on high‐throughput gene sequencing technology, No. 2022YFS0152.

## Supporting information


**Supporting Information** Additional supporting information can be found online in the Supporting Information section. The following supplementary materials are available online: Table S1: Summary of missing data for all variables included in the primary analysis. Table S2: Comparison of the discriminative ability for diabetic retinopathy between the albumin‐derived fructosamine (AlbF) ratio and its separate components. Table S3: Variance inflation factors (VIFs) for assessing multicollinearity among exposure variables and key covariates. Table S4: Results of sensitivity analysis by adding other potential covariates to the fully adjusted model (Model 4). Figure S1: Receiver operating characteristic (ROC) curves comparing the discriminative performance for diabetic retinopathy among four different exposure models. Figure S2: Correlation matrix diagram illustrating the relationships between all variables included in the study. Figure S3: Restricted cubic spline plot showing the nonlinear association between AlbF levels and the odds of diabetic retinopathy (with four knots). Figure S4: Binned residual plot for evaluating the goodness‐of‐fit of the final logistic regression model. Figure S5: Influence diagnostics to identify potential outlier observations or high‐leverage points. Figure S6: Comprehensive assessment of multicollinearity among predictor variables. Figure S7: Receiver operating characteristic (ROC) curve evaluating the diagnostic performance of albumin‐derived fructosamine (AlbF) as a standalone biomarker for diabetic retinopathy.

## Data Availability

The data used in this study are available from the corresponding author on reasonable request.
