# Supplementary material for: A Study of the Relationship Between Serum Albumin–Corrected Fructosamine and Type 2 Diabetic Retinopathy
Source: J Diabetes Res. 2026 Feb 2;2026:9275699. doi: 10.1155/jdr/9275699 (PMC12865125; doi:10.1155/jdr/9275699)
Supplement: Supplementary file 1 — Supporting Information Additional supporting information can be found online in the Supporting Information section. The following supplementary materials are available online: Table S1: Summary of missing data for all variables included in the primary analysis. Table S2: Comparison of the discriminative ability for diabetic retinopathy between the albumin‐derived fructosamine (AlbF) ratio and its separate components. Table S3: Variance inflation factors (VIFs) for assessing multicollinearity among exposure variables and key covariates. Table S4: Results of sensitivity analysis by adding other potential covariates to the fully adjusted model (Model 4). Figure S1: Receiver operating characteristic (ROC) curves comparing the discriminative performance for diabetic retinopathy among four different exposure models. Figure S2: Correlation matrix diagram illustrating the relationships between all variables included in the study. Figure S3: Restricted cubic spline plot showing the nonlinear association between AlbF levels and the odds of diabetic retinopathy (with four knots). Figure S4: Binned residual plot for evaluating the goodness‐of‐fit of the final logistic regression model. Figure S5: Influence diagnostics to identify potential outlier observations or high‐leverage points. Figure S6: Comprehensive assessment of multicollinearity among predictor variables. Figure S7: Receiver operating characteristic (ROC) curve evaluating the diagnostic performance of albumin‐derived fructosamine (AlbF) as a standalone biomarker for diabetic retinopathy. [file JDR-2026-9275699-s001.docx]

****Supplementary Material Files****

To:**A study of the relationship between serum albumin-corrected fructosamine and type 2 diabetic retinopathy**

By Zejiang Liu, Qiyun Long, Xuhui Song, Qin Guo, Tao Li , Huaguo Wang, Xing Qi, Sheng Lin

Correspondence : Sheng Lin, Email:zxyyshenggege@163.com, ORCID: 0009-0009-3393-4284

**Supplement Table S1** **Missing-data summary for all variables included in the analysis.**

****Supplement** Table S2** Discriminative ability for diabetic retinopathy: AlbF ratio versus separate components

****Supplement** Table S3** **Variance-inflation factors for exposure variables and key covariates**

**Supplement Table S4** sensitivity analysis (Added other covariates to Model4).

****Supplement Figure S1**** ROC curves comparing discriminative performance for diabetic retinopathy among four exposure models.

****Supplement Figure S2**** Correlation Matrix Diagram.

****Supplement Figure S3**** Restricted cubic spline for AlbF and diabetic retinopathy (4 knots).

****Supplement Figure S4****. Binned residual plot for the final logistic model.

****Supplement Figure S5**** Influence diagnostics.

****Supplement Figure S6**** Multicollinearity assessment.

****Supplement Figure S7****. Receiver Operating Characteristic (ROC) curve for Albumin-derived Fructosamine (AlbF) as a biomarker for diabetic retinopathy.

**Supplement Table S1** **Missing-data summary for all variables included in the analysis**

| Variable | Miss.freq | Miss.percentage% |
| --- | --- | --- |
| Apolipoprotein A | 1 | 0.241 |
| Apolipoprotein B | 1 | 0.241 |
| HDL | 1 | 0.241 |
| LDL | 1 | 0.241 |
| Total cholesterol | 1 | 0.241 |
| Triglycerides | 1 | 0.241 |
| Fructosamine | 2 | 0.4819 |
| Glucose | 2 | 0.4819 |
| Glycohemoglobin | 2 | 0.4819 |
| Weight | 8 | 1.9277 |
| BMI | 63 | 15.1807 |
| Height | 63 | 15.1807 |
| HTN meds | 206 | 49.6386 |
| HTN duration | 245 | 59.0361 |

Abbreviations: HDL-C: High density lipoproteincholesterol; LDL-C: Low-Density Lipoprotein Cholesterol; BMI, Body Mass Index; HTN, hypertension; HTN duration, years since diagnosis; HTN meds, use of antihypertensive medication.

****Supplement** Table S2** Discriminative ability for diabetic retinopathy: AlbF ratio versus separate components

| Model | Variable | AUC | 95%CI |
| --- | --- | --- | --- |
| Model 1 | AlbF | 0.69 | 0.64 - 0.74 |
| Model 2 | Fructosamine | 0.63 | 0.58 - 0.68 |
| Model 3 | Albumin | 0.64 | 0.58 - 0.69 |
| Model 4 | Fructosamine + Albumin | 0.69 | 0.64 - 0.75 |

•Abbreviations: AUC, the area under the ROC curve; CI, confidence interval; AlbF, Fructosamine/Albumin.

****Supplement** Table S3** **Variance-inflation factors for exposure variables and key covariates**

| Variable | Change percentage | VIF | colinearity |
| --- | --- | --- | --- |
| Crude | Ref | 120.863 | 1 |
| Age | -48.2 | 1.244 | 0 |
| Gender | -0.8 | **2.15** | 0 |
| DM duration | 8.4 | 1.293 | 0 |
| DM meds | -16.4 | 1.156 | 0 |
| BMI | 5.2 | 1.197 | 0 |
| Glycated haemoglobin | -6.5 | 1.936 | 0 |
| Creatinine | 1.8 | 1.61 | 0 |
| Total cholesterol | 1.8 | 1.323 | 0 |
| Triglycerides | 1.2 | 1.346 | 0 |
| Lymphocyte | -1.1 | 1.202 | 0 |
| Haemoglobin | 0 | 1.728 | 0 |
| Albumin | -58.5 | 21.03 | 1 |
| Fructosamine | -70.8 | 105.725 | 1 |

Abbreviations: DM, diabetes mellitus; DM duration, years since diagnosis; DM meds, use of glucose-lowering medication; BMI, Body Mass Index.

**Supplementary Table S4** sensitivity analysis (Added other covariates to Model4).

| Variable | n.total | n.event % | Model4 | | Model5a | | Model5b | | Model5c | |
| --- | --- | --- | --- | --- | --- | --- | --- | --- | --- | --- |
|  |  |  | adj.OR (95%CI) | adj *P* | adj.OR (95%CI) | adj *P* | adj.OR (95%CI) | adj *P* | adj.OR (95%CI) | adj *P* |
| AlbF, per 10 μmol/g* | 415 | 174 (41.9) | 1.88 (1.36~2.6) | <0.001 | 1.92 (1.38~2.66) | <0.001 | 1.92 (1.38~2.66) | <0.001 | 1.85 (1.33~2.58) | <0.001 |
| AlbF category (per 10 μmol/g) | | | | | | | | | | |
| T1 (low) | 138 | 27 (19.6) | 1(Ref) |  | 1(Ref) |  | 1(Ref) |  | 1(Ref) |  |
| T2 | 138 | 68 (49.3) | 5.5 (2.53~11.96) | <0.001 | 5.4 (2.48~11.78) | <0.001 | 5.41 (2.47~11.82) | <0.001 | 4.88 (2.21~10.77) | <0.001 |
| T3 (high) | 139 | 79 (56.8) | 7.2 (2.82~18.4) | <0.001 | 7.27 (2.84~18.63) | <0.001 | 7.41 (2.86~19.19) | <0.001 | 6.55 (2.49~17.22) | <0.001 |
| *p* for trend |  |  |  | <0.001 |  | <0.001 |  | <0.001 |  | <0.001 |

*AlbF was entered as a continuous variable scaled per 10 umol/g to facilitate interpretation.

Model4: age, gender, DM duration, DM meds, Hypertension, BMI, Glycated haemoglobin, Creatinine, Total cholesterol, Triglycerides, Lymphocyte, Haemoglobin;

Model 5a: Model 4 + Smoking, Drinking;

Model 5b: Model 4 + Smoking, Drinking, HDL, LDL;

Model 5c: Model 4 + Smoking, Drinking, HDL, LDL, Apolipoprotein A, Apolipoprotein B.

Abbreviations: BMI, Body Mass Index; AlbF. Fructosamine/Albumin; DM, diabetes mellitus; DM duration, years since diagnosis; DM meds, use of glucose-lowering or antihypertensive medication; HDL-C, High density lipoproteincholesterol; LDL-C, Low-Density Lipoprotein Cholesterol, OR, Odds Ratio; CI, Confidence Interval.


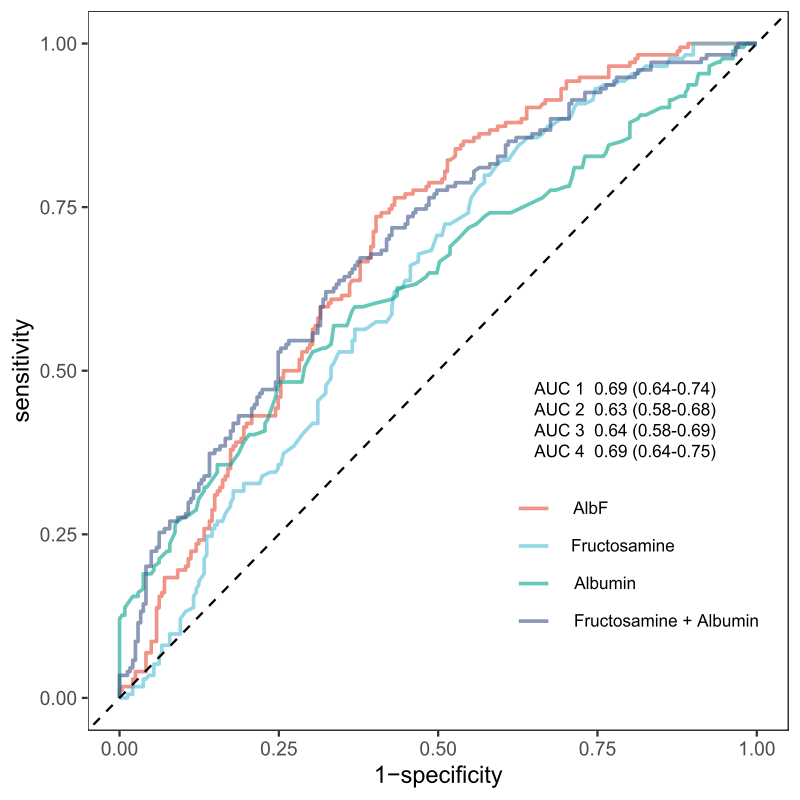


****Supplement Figure S1**** ROC curves comparing discriminative performance for diabetic retinopathy among four exposure models. Abbreviations: AlbF, Fructosamine/Albumin.

****
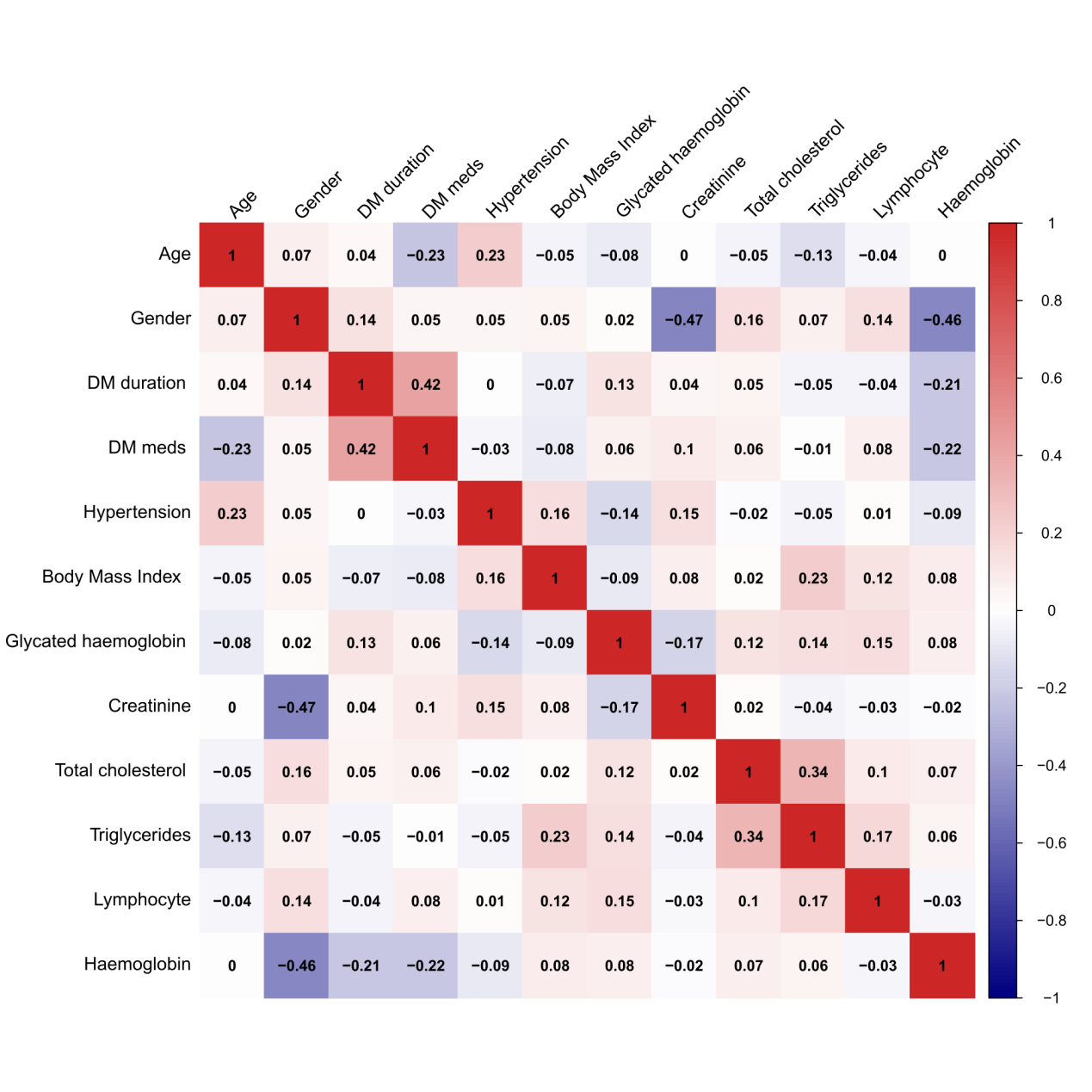
****

****Supplement Figure S2**** Correlation Matrix Diagram. Abbreviations: DM, diabetes mellitus; DM duration, years since diagnosis; DM meds, use of glucose-lowering medication.


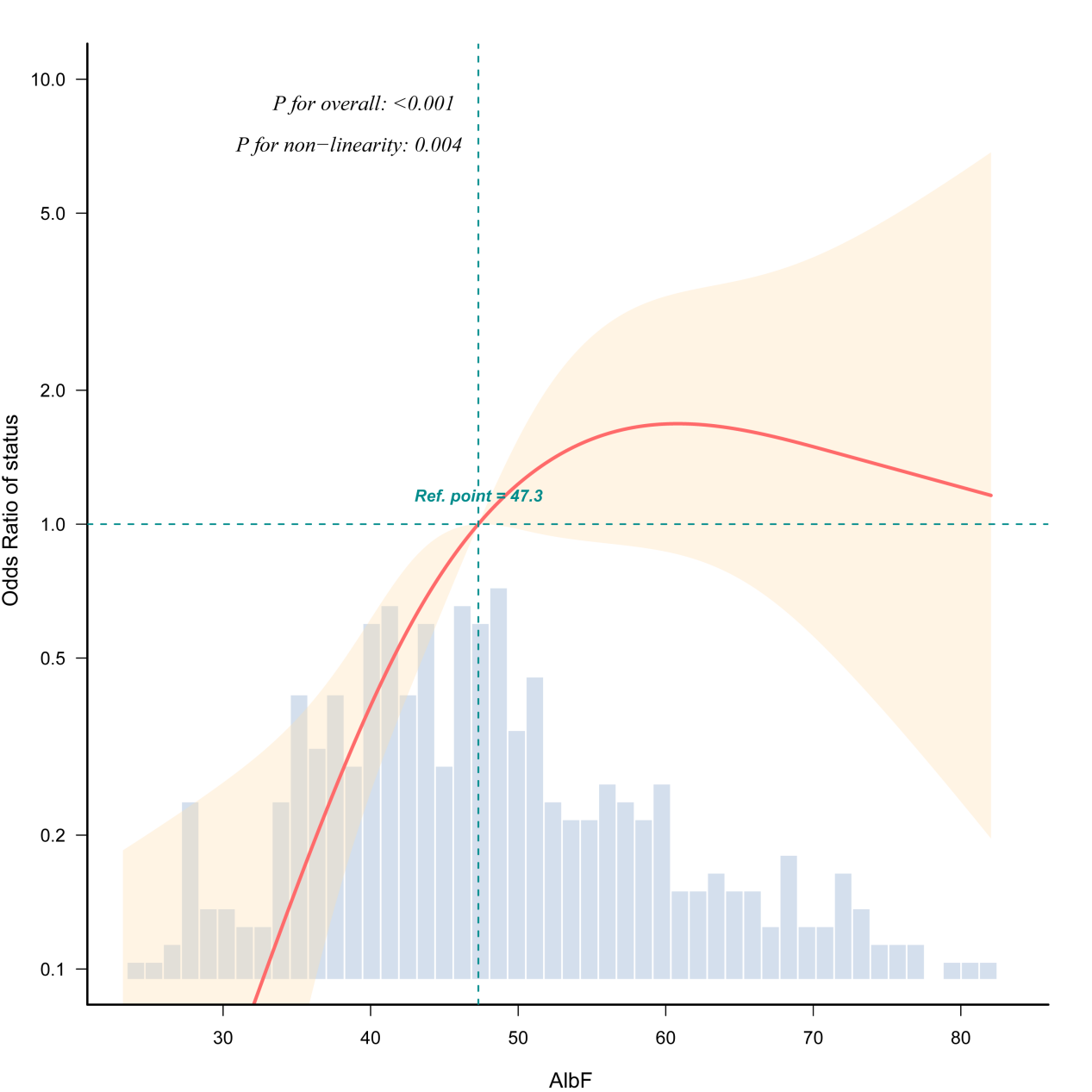


****Supplement Figure S3**** Restricted cubic spline for AlbF and diabetic retinopathy (4 knots). To reduce sparse-data influence, the x-axis was truncated at the 99th percentile. All odds ratios were adjusted for the full set of covariates ( age, gender, DM duration, DM meds, Hypertension, BMI, Glycated haemoglobin, Creatinine, Total cholesterol, Triglycerides, Lymphocyte, Haemoglobin ).

Abbreviations: DM, diabetes mellitus; AlbF, Fructosamine/Albumin; DM duration, years since diagnosis; DM meds, use of glucose-lowering medication, BMI, Body Mass Index.


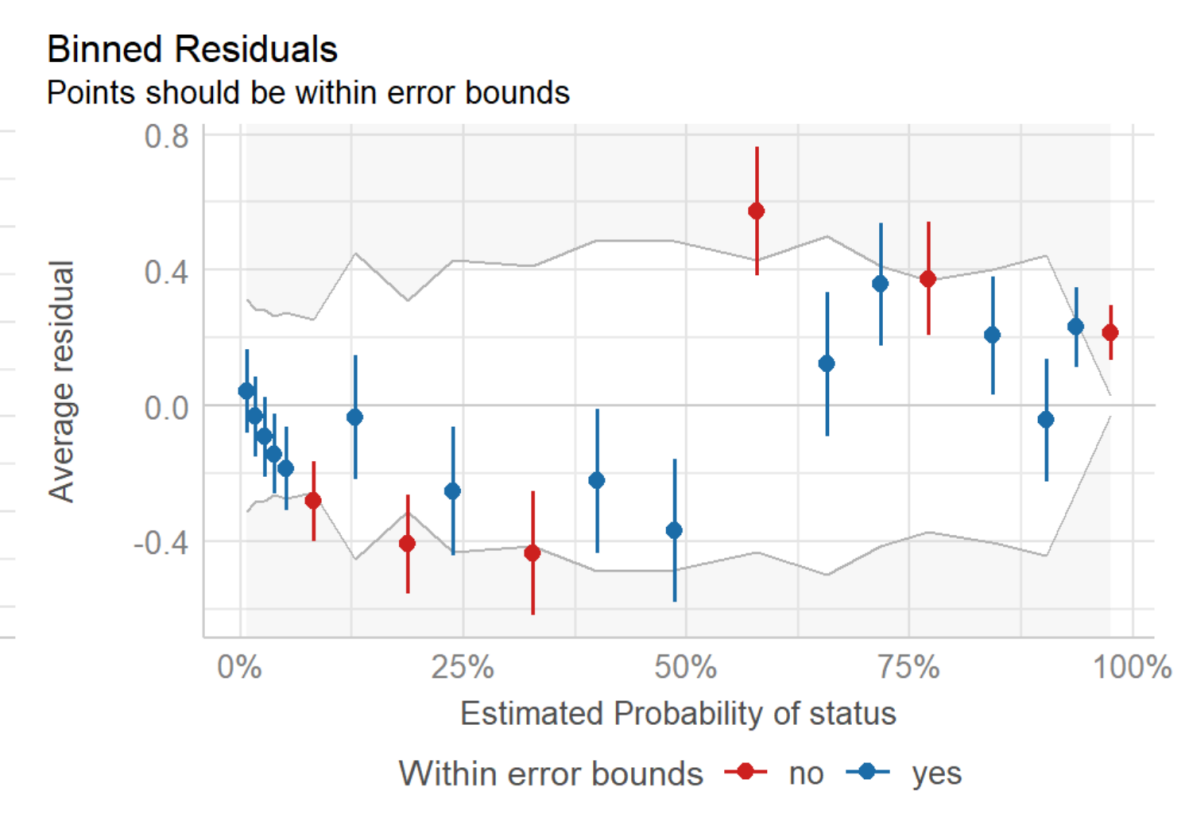


****Supplement Figure S4****. Binned residual plot for the final logistic model.
Blue dots = eyes without DR (observed − expected proportion); red dots = eyes with DR. Vertical bars = ±2-SE error bounds. All points lie within the 95 % envelope, indicating good calibration.


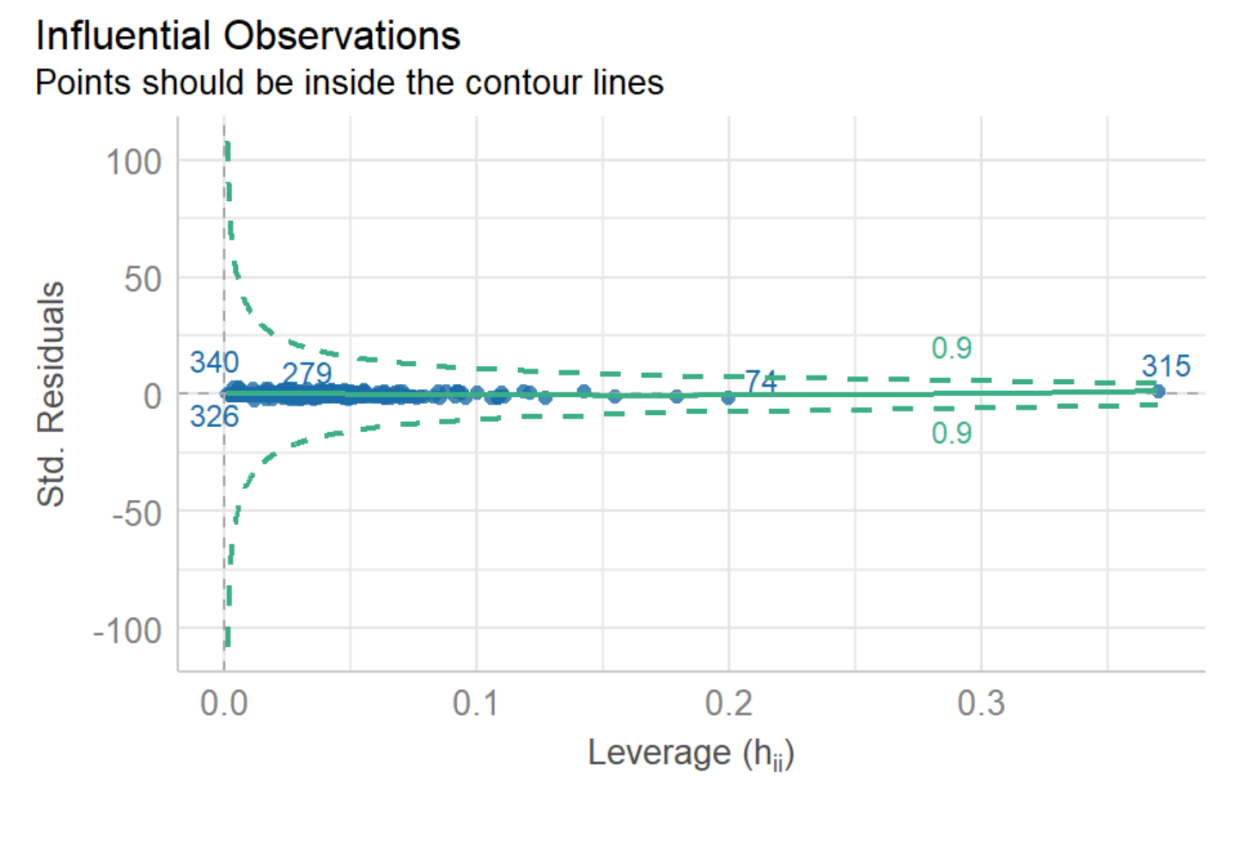


****Supplement Figure S5****. Influence diagnostics. Leverage (h) versus standardized Pearson residuals with Cook’s distance contours (0.5 and 1.0). No observation exceeds the Cook’s D = 1 boundary, demonstrating the absence of influential cases.


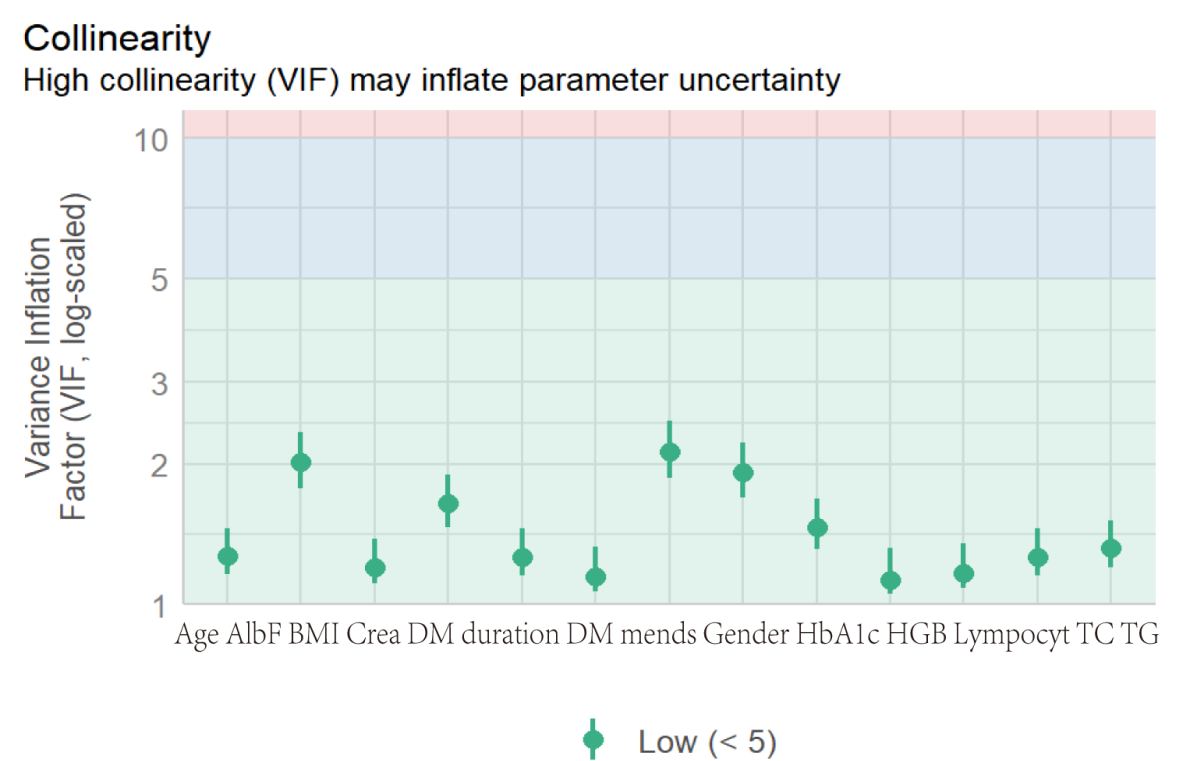


****Supplement Figure S6****. Multicollinearity assessment. Variance Inflation Factor (VIF) for each covariate in the full model. The dashed line marks VIF = 5; all values remain below this threshold, confirming low multicollinearity.


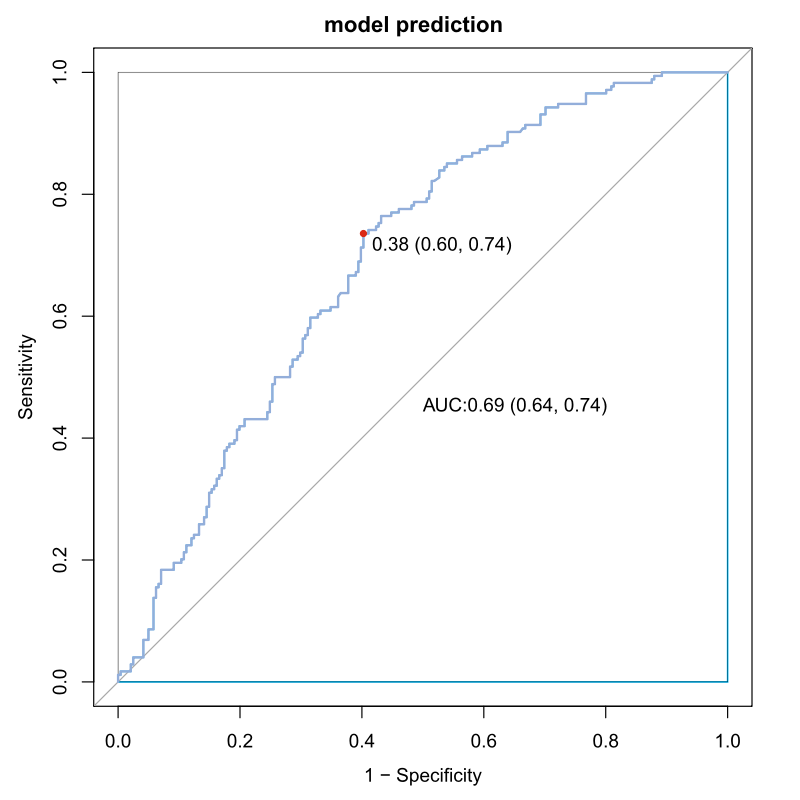


**Supplement Figure S7.** Receiver Operating Characteristic (ROC) curve for Albumin-derived Fructosamine (AlbF) as a biomarker for diabetic retinopathy.

This curve assesses the ability of AlbF to identify individuals with diabetic retinopathy. The Area Under the Curve (AUC) of 0.69 (95% Confidence Interval [CI]: 0.64-0.74) indicates a fair diagnostic accuracy. The solid circle highlights the optimal prognostic cut-off value of 38.0 μmol/g, determined by Youden's index (J = 0.34), which yields a sensitivity of 74% and a specificity of 60%. This threshold represents a potential candidate for clinical use, particularly in scenarios where HbA1c is unreliable. The diagonal line (AUC = 0.50) serves as the reference for a test with no predictive power.
